# Not Led by the Nose: Volatiles from Undamaged *Eucalyptus* Hosts Do Not Influence Psyllid Orientation

**DOI:** 10.3390/insects9040166

**Published:** 2018-11-17

**Authors:** Kevin Farnier, Noel W. Davies, Martin J. Steinbauer

**Affiliations:** 1 Department of Ecology, Environment and Evolution, La Trobe University, Melbourne, VIC 3086, Australia; m.steinbauer@latrobe.edu.au; 2 Department of Economic Development, Jobs, Transports and Resources, Agriculture Victoria Research, Melbourne, VIC 3083, Australia; 3 Central Science Laboratory, University of Tasmania, Hobart, TAS 7001, Australia; noel.davies@utas.edu.au

**Keywords:** *Eucalyptus*, Aphalaridae, Jumping plantlice, sensory ecology, terpenes, foraging kairomone

## Abstract

Psyllids (Hemiptera: Psylloidea) are small sucking insects with high host plant specificity. Despite the primitive olfactory system of psyllids, some species have been suggested to rely on host plant volatiles (HPVs) for seasonal migration between summer deciduous hosts and winter coniferous hosts. Similarly, enhanced attraction of psyllid vectors has been observed as a result of the manipulation of host odors by plant pathogens. As yet, there are no studies of olfaction in psyllids that utilize evergreen eucalypt hosts. We investigated the behavioral responses of adults of four *Eucalyptus*-feeding psyllids—*Ctenarytaina eucalypti*, *C. bipartita*, *Anoeconeossa bundoorensis* and *Glycaspis brimblecombei*—to their respective HPVs in Y-tube olfactometer bioassays. We also used existing physiological data for *C. eucalypti* to investigate potential olfactory tuning that may modulate the preference for morphologically juvenile leaves over morphologically adult leaves. Although adult *C. eucalypti* were consistently repelled by HPVs from damaged host leaves, none of the species exhibited positive chemotaxis to HPVs from undamaged leaves. Surprisingly, *G. brimblecombei* was repelled by HPVs from undamaged host leaves. Our findings provide little support for a significant role of olfaction in host location by *Eucalyptus*-feeding psyllids. We propose a number of ecological hypotheses to explain these unexpected findings.

## 1. Introduction

With approximately 3850 described species and probably as many undescribed species, psyllids (Hemiptera: Psylloidea) are a globally diverse and highly successful taxon [1]. Unlike aphids, their close sternorrhynchan allies, psyllids are thought to originate from a highly specialized monophyletic lineage which may explain the prevalence of mono- or oligophagy. Even though psyllids have close associations with their hosts, poor synchrony between their evolution and that of their host suggests that speciation has been driven by sequential sympatric colonization of plants via “host-switching events” rather than via reciprocal co-evolution [2,3,4,5]. Nevertheless, case studies have demonstrated that some host-switching events may have resulted in the evolution of detoxification mechanisms to specific plant secondary metabolites [4,6,7]. Interestingly, psyllid colonies bred on different hosts for as few as 12 generations have been shown to already exhibit differences in their detoxification enzymes [8]. At the single generation scale, greater offspring survival and fitness were observed as a result of retained oviposition preferences for the maternal host [9]. Since very little is known about psyllid senses, it is difficult to know how they might explain host-switches or whether they have changed in response to long periods of association with novel hosts. 

In aphids, which have been more extensively studied than psyllids, host selection comprises two important behavioral phases [10,11,12,13,14]. The first phase is “host-finding” which is initiated when the insect leaves a plant and ends when it lands on a new one. Host-finding involves a succession of dispersal and attacking flights between nearby plants mediated by pre-alighting olfactory and visual cues. The second phase is “host selection” and encompasses behaviors that occur after landing. Olfactory, mechanical and/or gustatory cues perceived during selection determine whether the insect “settles” and initiates sustained feeding and oviposition (acceptance) or whether it re-engages in host finding (rejection). Visual cues such as leaf shape, size and color or even light polarization have been identified as important factors in host finding by some psyllids [15,16,17,18,19,20]. The significance of plant-specific and presumably more reliable olfactory cues provided by HPVs remains uncertain [21,22].

Morphological, neuroanatomical and physiological studies of a limited number of psyllid species have revealed a rudimentary olfactory system, probably best adapted to the detection of a limited number of common terpenoids, aldehydes and alcohols comprising the headspaces of psyllid hosts [23,24,25,26,27,28]. In the carrot psyllid (*Trioza apicalis*), different classes of olfactory receptor neuron were found to be specific to few key compounds present both in winter (conifer) and summer hosts (carrot) [26]. Among them was a carrot-specific sesquiterpene, putatively identified as bergamotene, believed to mediate psyllid preference for their summer host [29]. Similar observations were reported in another host alternating psyllid species, namely the apple psyllid (*Cacopsylla picta*). This psyllid switches between deciduous summer (apple and hawthorn) and coniferous winter hosts (pine and spruce) which exhibit key similarities in HPV profiles [30]. In these examples, the specificity of psyllid olfactory systems has been suggested to be the product of adaptive trade-offs resulting in lower detection thresholds for a few key volatiles that facilitate seasonal migration.

Increased emissions of *β*-caryophyllene in apple tree volatiles infected by Phytoplasma, have been shown to considerably enhance the attraction of *C. picta—*the psyllid species that the phytopathogen uses as vector to colonize new trees [31,32,33]. In a similar way, the Asian citrus psyllid (*Diaphorina citri*) has been reported to prefer HPVs from hosts infected with *Candidatus* Liberibacter asiaticus over healthy hosts due to the emission of methyl salicylate in their bouquet of volatiles [34,35,36]. In both cases, attractions to modified HPVs via pathogen intervention appear to be deceptive and are therefore unlikely to be adaptive. 

**Figure 1:**
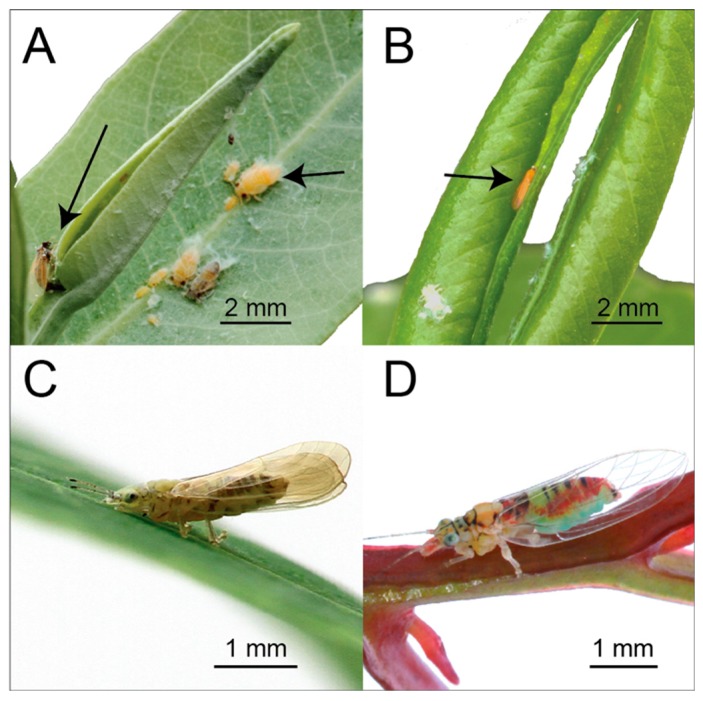
(**A**) Adult *C. eucalypti* (extreme left) ovipositing into the crevice of the apical bud of *E. globulus*; nymphs (right) on the first expanding leaf pair. (**B**) Adult *C. bipartita* inside the distorted apical bud of *E. kitsoniana*; distortion of leaves into a leaf roll is induced by nymphal feeding. (**C**) Adult *A. bundoorensis*. (**D**) Adult *G. brimblecombei*.

In this study, we focussed on four co-occurring Australian native psyllids species. *Ctenarytaina eucalypti* and *C. bipartita* live exclusively in the closed apical buds, characteristic of the juvenile (sessile) foliage of their heterophyllous hosts—*Eucalyptus globulus* and *E. kitsoniana*, respectively [37,38,39]. The morphology of the buds provides the tight crevices into which females oviposit and provide sheltered microhabitats that protect nymphs from desiccation (Figure 1A,B). The disappearance of suitable microhabitats associated with the subsequent production of the petiolate—adult leaves by host trees as they mature—provides the main impetus for dispersal between hosts. Since apical buds are small and obscured by expanded juvenile leaves, host finding by adults after landing is considered likely. The protected morphology of apical buds presumably exposes adults and nymphs to highly concentrated odor microhabitats. *Anoeconeossa bundoorensis* is a recently described species, the exact host range and geographical distribution of which is unknown [40]. Adults and nymphs feed on young leaves but adults appear to oviposit in depressions on young stems of *E. camaldulensis* necessitating that nymphs disperse after hatching (Figure 1C). Unlike the two *Ctenarytaina* species, adults are active foragers that exhibit prolonged bouts of walking on stems and leaves. The lerp-forming species *Glycaspis brimblecombei* (i.e., nymphs develop under a cover made from their anal excretions) utilizes *E. camaldulensis* as primary host but is also known to occur on a wider range of *Eucalyptus* species from the subgenus *Symphyomyrtus.* Nymphs and adults feed and oviposit on adaxial leaf surfaces (Figure 1D)*.* Australian psyllids account for >10% of psyllid species globally and approximately 90% of them are specific for *Eucalyptus* [41,42]. With their unique diversity and high degree of specificity for closely related hosts, Australian psyllids constitute ideal models to investigate potential sensory adaptations in psyllid species relying entirely on evergreen hosts.

The orientation responses of our model species in the presence of their respective HPVs were tested in a Y-tube olfactometer. Inter- and intraspecific (latter in the case of *E. globulus*) differences in HPVs were examined using dynamic headspace sampling and GC-MS analysis. An evaluation of potential olfactory tuning to key components susceptible to facilitate discrimination between juvenile and adult host leaves (*E. globulus*) in *C. eucalypti* was conducted by fitting host volatiles analysis results with existing physiological (Single Sensillum Recording) data [27]. We use our results to re-define the relative contribution of olfaction and other sensory modalities in the host location and selection processes of these species.

## 2. Materials and Methods 

### 2.1. Trees and Insects

Colonies of *C. eucalypti*, *C. bipartita* and *A. bundoorensis*, started from individuals collected from La Trobe University (Bundoora campus, Melbourne, Australia) and Fish Creek (Victoria, Australia), were all maintained on potted hosts (30 cm diameter black plastic pots) in separate 90 cm × 90 cm × 90 cm mesh cages in the same temperature-controlled glasshouse (constant 24 °C). In the absence of a lighting system, trees were subjected to a natural day/night light cycle throughout the year. Watering regime was adjusted according to seasonal changes in weather conditions. *Eucalyptus* trees used for Y-tube experiments, volatile collections and for maintaining psyllid colonies were either grown from seeds obtained from the Australian Tree Seed Bank (CSIRO, Canberra, Australia) or purchased as seedlings from a local plant nursery (Wimmera Native Nursery, Dimboola, Victoria, Australia). Trees used in Y-tube experiments were transported from the glasshouse to the lab shortly before experiments and returned to the glasshouse at the end the day. Different trees were used every experimental day to account for possible intraspecific variation between different tree odors. Since the lerp-forming species *G. brimblecombei* could not be reared in cages, insects of this species were collected on the days of experiments from infested *E. camaldulensis* trees located at La Trobe University (Melbourne, Australia). Psyllids used in Y-tube experiments were collected on the mornings before experiments and starved for a minimum of two hours before testing. 

### 2.2. Behavioral Responses

**Figure 2:**
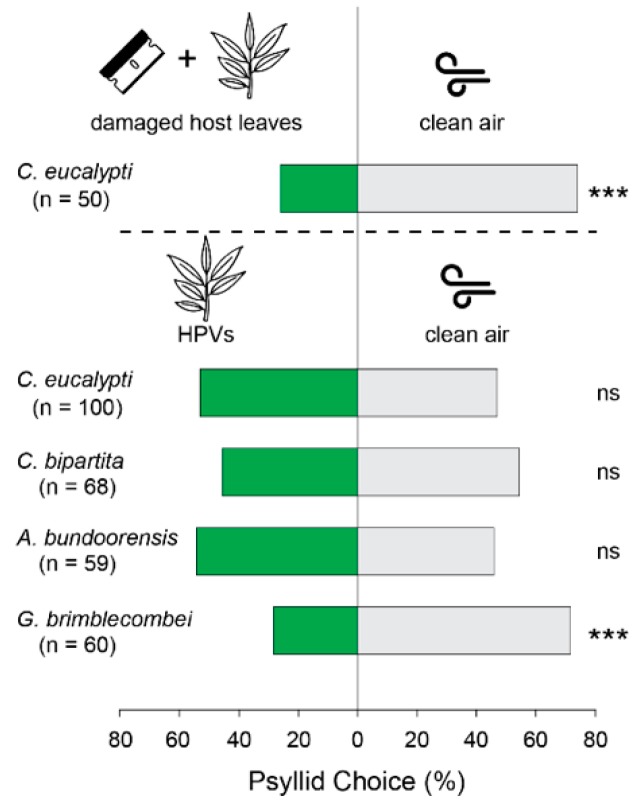
Psyllid responses in the Y-tube olfactometer. Bars above dashed lines show the results of a pilot experiment using damaged *E. globulus* juvenile leaves as ‘negative control’ to confirm psyllids’ ability to orient in response to odor stimuli under our experimental conditions. Green bars represent attraction to HPVs and grey bars attraction to clean air. (*** = *p* < 0.001; ns = not significant). ‘n’ under species’ names indicates the number of responders in each experiment.

Experiments were conducted between 10:00 and 19:00 during the Austral spring-summer-autumn (from October to May)—psyllids are most active during this period. *C. eucalypti* and *C. bipartita* were tested during spring and autumn when their populations are at their highest whereas *A. bundoorensis* and *G. brimblecombei* were tested in summer (from December to February). Psyllid behavioral responses were tested in a Y-tube glass olfactometer (8 cm long test arms, 12 cm-middle arm, 1.5 cm inner diameter) placed on a white Perspex bench inclined vertically at 30°, inside a laboratory chamber surrounded by white fabric curtain under light-controlled conditions [15]. Four 28 W/865 UV+ fluorescent light tubes (Philips, Holland, The Netherlands) placed on top of the chamber were mounted on light units fitted with custom-made high frequency electronic dimmable ballasts (1.2 kHz) providing illumination conditions comparable to natural conditions. Light was homogenized by a sheet of Rosco 216 (UV-permitting) white diffusion screen (Rosco, Munich, Germany) and the irradiance adjusted at 50 µmol·m^−2^·s^−1^ (~2200 lux). The position of the olfactometer on the bench was adjusted to ensure equal light intensity between both arms of the Y-tube which was verified by means of a Fieldscout Quantum lightmeter (Spectrum Technologies, Inc., Aurora, IL, USA). Host volatiles emanating from branchlets comprising a minimum of five young intact leaf pairs selected from the apical end (typically 25–30 cm in length) were confined in oven bags (Multix, Glen Waverley, Australia 30 × 38 cm) and entrained in a 250 mL min^−1^ purified and humidified airflow connected to one arm of the olfactometer while a similar airflow was applied to an empty oven bag connected to the control arm of the olfactometer. Airflow entering each arm of the olfactometer was controlled with a digital airflow meter and the system was left to equilibrate for 10–30 min prior to testing. Psyllids in individual containers were anesthetized in a −20 °C freezer for 4 min and subsequently placed one at a time in the main stem of the Y-tube using a fine paint brush. The orientation of individual psyllids was observed for 30 min when simultaneously exposed to host volatiles (treatment arm) and clean air (control arm). A choice was considered to have been made when a psyllid reached the middle of either test arm (4 cm inside the arm) which, in most instances, corresponded to a non-return point. Psyllids that did not choose within 30 min were considered as “non-responding” and excluded from the dataset. Likewise, individuals that exhibited erratic behaviors, or came to rest in an arm of the olfactometer either by jumping and/or bouncing off the walls of the olfactometer were discarded. A minimum of 50 insects were tested in each experiment. Males and females were tested in separate olfactometers and odor sources were swapped every four insects to prevent any positional bias. The olfactometer was left to equilibrate for 10 min after each odor source swap, before the next insect was released. At the end of the experiments, Y-tubes were soaked in warm soapy water, then rinsed in cold clear water and subsequently baked overnight in an oven at 280 °C. In the absence of a positive control, the ability of psyllids to respond to odors under our experimental conditions was verified with consistent aversion to the odor of mechanically damaged host leaves exhibited by *C. eucalypti* in pilot experiments (Figure 2).

### 2.3. Host Plant Volatiles Collection and Analysis

Host branchlets (five to six leaf pairs) of *Eucalyptus globulus* (juvenile and adult foliage), *E. kitsoniana* and *E. camaldulensis* were enveloped in oven bags in a similar way as described in the Y-tube experiments. Volatiles were collected by applying a purified and humidified airflow on one side of the bag at 700 mL·min^−1^ while a similar airflow was pulled through adsorbent filters (50 mg, Super Q, Alltech Inc., Deerfield, IL, USA) for 8 h. Analytes were eluted from filters with 200 μL of dichloromethane. Chemical analysis was performed using a Varian 3800 GC gas chromatograph equipped with a VF-5MS column (30 m × 0.25 mm × 0.25 μm) coupled with a Bruker 300 triple quadrupole mass spectrometer. Helium was used as carrier gas in constant flow mode (1.2 mL·min^−1^). A 2 µL aliquot of the extracts was injected by means of a Varian CP-8400 autosampler in split mode (4:1) at 220 °C. The initial oven temperature was 40 °C, held for 2 min, subsequently increased to 225 °C at 8 °C·min^−1^. Quantification was performed in ‘tridecane equivalents’ ng per hour via the comparison of peak areas of different compounds with that of an internal (5 μg of n-tridecane) added to the extracts. A mass spectrometer was operated in EI mode (70 eV) with the scan range set between *m*/*z* 35 and 350 at 1.2 scans per second. Compounds were tentatively identified based on their mass spectrum by comparison with the NIST08 mass spectral library and an ‘in-house’ library of essential oil compounds, and, where possible, by comparison of Kovats retention indices with those of synthetic compounds [43].

### 2.4. Statistical Analyses

All statistical analyses were performed using Past version 3.02 [44] and SPSS statistics (v23, IBM, New York, NY, USA). Psyllid responses in the Y-tube olfactometer were tested using non-parametric binomial tests posing an equal probability for psyllids to choose between the odor-treated and control arm as null hypothesis. Intra- and interspecific differences in total volatiles emissions were analyzed using non-parametric Kruskall–Wallis analysis of variance followed by Mann–Whitney tests with Sidak corrections for multiple pairwise comparisons. The chemical profiles of different *Eucalyptus* hosts headspaces were compared by one-way ANOSIM using Bray–Curtis dissimilarity matrix. Only volatiles that accounted for, on average, at least 1% of the chemical profile of any *Eucalyptus* species were included in the analysis. SIMPER analysis was used to determine the relative contribution of different compounds to the dissimilarity between the headspace of different species. A similar approach was employed to compare the volatile profiles of adult and juvenile leaves of *E. globulus* as a whole as opposed to as perceived by psyllids olfactory system. This was achieved by conducting a similar analysis but this time including only volatiles that elicited antennal responses in SSR studies conducted with *C. eucalypti* [27], namely: *β*-myrcene, *cis*-3-hexenyl acetate, *trans*-*β*-ocimene, linalool, *β*-caryophyllene. 

## 3. Results

### 3.1. Behavioral Responses

Psyllid responsiveness in the Y-tube was 77% for *C. eucalypti*, 74% for *C. bipartita*, 70% for *A. bundoorensis* and 69% for *G. brimblecombei*. No significant preference between male and female choice was observed in all species and therefore, data from both sexes were pooled. Surprisingly, none of the four species were attracted to their host odors; *C. eucalypti* (*p = 0.76*), *C. bipartita* (*p* = 0.27), *A. bundoorensis* (*p* = 0.78). Even more surprising was the behavior of *G. brimblecombei* which strongly avoided host plant odors (*p* < 0.001, Figure 2).

### 3.2. Host Plant Volatiles

**Figure 3:**
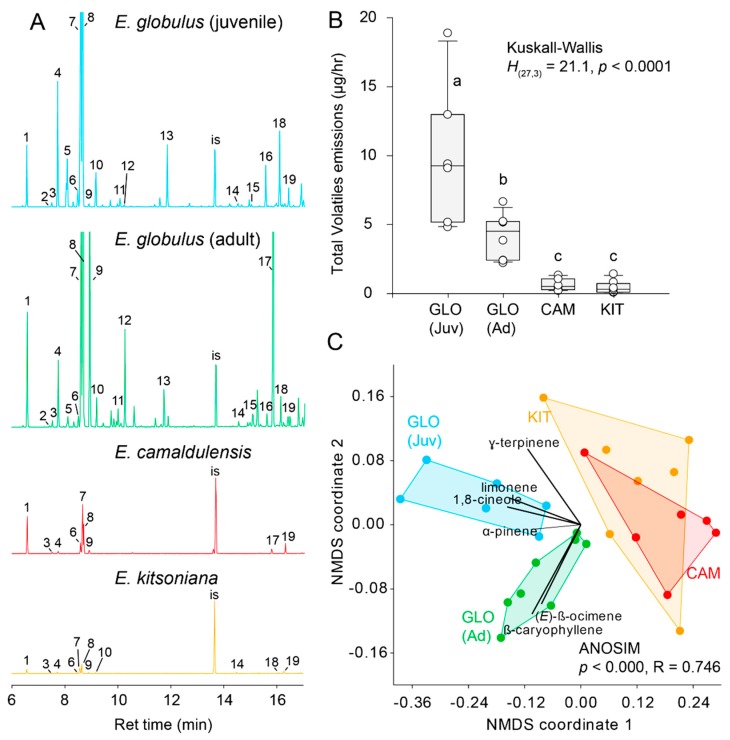
(**A**) Chromatograms of HPVs of leaves of *E. globulus* (juvenile and adult), and young *E. camaldulensis* and *E. kistoniana* leaves. Numbers correspond to the main components of the headspace. Key is as follows: 1. *α*-pinene, 2. sabinene, 3. *β*-pinene, 4. *β*-myrcene, 5. *cis*-3-hexenyl acetate, 6. p-cymene, 7. limonene, 8. 1,8-cineole, 9. *β*-ocimene, 10. *γ*-terpinene, 11. linalool, 12. DMNT, 13. *α*-terpineol, 14. terpinyl acetate, 15. *α*-copaene, 16. *α*-gurjunene, 17. *β*-caryophyllene, 18. aromadendrene, 19. alloaromadendrene, IS. internal standard (tridecane). (**B**) Boxplot depicting quantitative differences in volatiles emitted by different *Eucalyptus* species; letters indicate statistical differences. (**C**) NMDS ordination of *Eucalyptus* HPV profiles (ordination based on Bray–Curtis dissimilarity matrix); GLO (Ad) = adult *E. globulus*, GLO (Juv) = juvenile *E. globulus*, KIT = *E. kitsoniana* and CAM = *E. camaldulensis*.

Results of GC-MS analyses (Table 1) revealed significant qualitative (Figure 3A) and quantitative (Kruskall–Wallis *H*_(27,3)_ = 21.2, *p* < 0.001, Figure 3B) differences between the volatiles profiles of different *Eucalyptus* hosts. The quantity of volatiles emitted by *E. globulus* leaves was up to 20-times greater than that of *E. camaldulensis* and *E. kitsoniana* from which comparable amounts of volatiles emanated (Mann–Whitney *U* = 49.0, *p* = 0.35). Juvenile *E. globulus* leaves produced significantly greater quantities of volatiles than adult leaves (Mann–Whitney *U* = 62.0, *p* = 0.03). 

Overall, the volatile profiles of all hosts differed significantly (one-way ANOSIM, *p* < 0.0001, R = 0.746, Figure 3C). 1,8-cineole (eucalyptol) alone contributed 70.2% of the dissimilarity between different host odors followed by *β*-ocimene (9.7%), limonene (8.5%) and terpinyl acetate (5.1%). *E. kistoniana* and *E. camaldulensis* exhibited the most similar odor profiles (*p* = 0.045); the latter emitted greater quantities of *α*-pinene, 1,8 cineole and *γ*-terpinene. Adult leaves of *E. globulus* had the most distinctive profile which was characterized by greater emissions of *β*-caryophyllene and *β*-ocimene (Figure 3A,C and Table 1).

**Figure 4:**
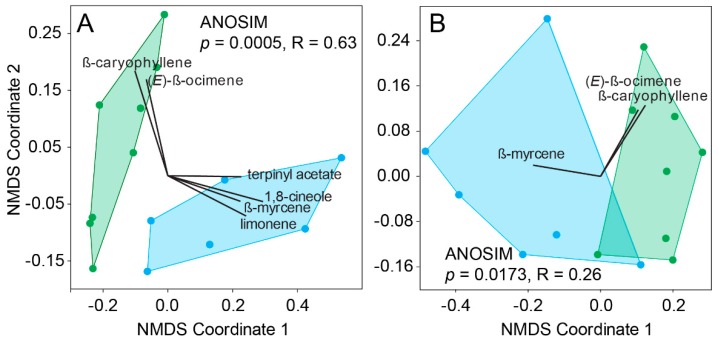
NMDS ordinations of the headspace chemical profiles of juvenile (in blue) and green (adult) foliage of the host *C. eucalypti* based on (**A**) the main constituents of the host headspace and (**B**) SSR-active compounds only.

The differences between the headspace of juvenile and adult leaves of *E. globulus* (ANOSIM, *p* = 0.0005, *R* = 0.63; Figure 4A) were due to the greater emissions of 1,8-cineole (35% of dissimilarity), limonene (14%) and terpinyl acetate (~4.5%) emitted by juvenile leaves. In contrast, *β*-ocimene (12.5%) and *β*-caryophyllene (5.7%) were more abundant in the emissions of adult leaves. A loss of resolution between the two headspace profiles was observed when only compounds detected by the antennae of *C. eucalypti* were considered (ANOSIM, *p* = 0.0173, *R* = 0.26; Figure 4B). Of all the compounds detected by *C. eucalypti*, *β*-ocimene (49%), *β*-caryophyllene (25%) and *β*-myrcene (20%) differed the most between the two leaf types while *cis*-3-hexenyl acetate (4.5%) and linalool (1.2%) were emitted in comparable amounts. Although the headspace profiles of the two leaf types remained significantly different when only psyllid-detected compounds were considered, the apparent loss of odor resolution suggests a lack of olfactory specialization toward an increased ability to discriminate between morphological leaf types.

## 4. Discussion

Despite their diversity and widespread distribution, the exact role played by olfaction and HPVs in psyllid dispersal and host location remain poorly understood in these highly specific herbivores. Despite marked differences in the chemistries of HPVs, none of the four psyllid species we tested exhibited positive chemotactic responses to host plant odors. Surprisingly, *G. brimblecombei* was even found to consistently avoid the odor of its host. While these results may appear surprising, they align with some of the existing literature concerning psyllid olfaction. Early studies of the citrus-feeding psyllid *Trioza erytrea* failed to observe orientation to host plant odors in various types of olfactometers [45,46]. In fact, attraction to host volatiles has only been observed in a limited number of psyllids species such as the pear (*Cacopsylla bidens*) [47] and Asian citrus psyllids (*D. citri*) [9,48,49,50]. However, in most cases, observed chemotactic responses were weak and often required the presence of visual stimuli [51]. To date, the most convincing examples of odor-mediated attraction involve phytopathogens which have apparently evolved the ability to attract psyllid vectors via the manipulation of HPV emissions. For instance, increased emissions of *β*-caryophyllene induced in apple trees infected with Phytoplasma which uses *C. picta* as a vector, were shown to elicit strong chemotactic responses in Y-tube olfactometer experiments [31,32,33]. In a similar way, deceptive attraction by hosts infected with *Candidatus* Liberibacter asiaticus has been reported in the Asian citrus psyllid (*Diaphorina citri*) via the emission of methyl salicylate in the volatile bouquet of infected plants [34,35,36]. However, such interactions are most likely peculiar and psyllid behavioral responses to manipulated host odors relate more to superstimuli rather than true host attraction. 

Psyllid behavior in our Y-tube olfactometer was characterized by occasional positive anemotaxis, ‘zig-zag’ movements in the main stem, short pauses at the intersection of both arms and subsequent resumption of movement toward the preferred arm. The capacity of psyllids to exhibit odor-mediated behaviors under our experimental conditions was confirmed by consistent aversion of *C. eucalypti* to high doses of volatiles from crushed host leaves. Aversion was also observed in *G. brimblecombei* but when exposed to the odors of undamaged host leaves. The latter behavior is difficult to explain considering the relatively low quantities of volatiles emitted by *E. camaldulensis* as well as the fact that *A. bundoorensis*, which shares the same host, was not deterred by host odors. Intriguingly, *G. brimblecombei* did not successfully breed on host trees maintained in the glasshouse as opposed to *A. bundoorensis* which reproduced in high numbers. While pre-concentration during the confinement of host volatiles in oven bags and different odor tolerance threshold between both species may have caused *G. brimblecombei* to avoid *E. camaldulensis* odors, it is not clear whether such deterrence reflects an artificial bias induced by our experimental conditions or a true avoidance to alienated odors of hosts grown in artificial conditions.

Nonetheless, a central question relating to our work remains: why were psyllids not attracted to HPVs? Conceptual frameworks concerning host selection propose that bouquets of HPVs provide specific and therefore reliable cues to insects for host location [21]. However, whether psyllids really engage in host location is unclear. Psyllids are essentially sedentary and leave their host to escape predators or when they no longer provide suitable food or oviposition sites [52,53]. Studies of the dispersal abilities of individual insects in release and re-capture experiments using *D. citri* have yielded inconsistent results ranging from distances from 5–12 m [54] up to 300 m [55]. The predominance of downwind movement in wind tunnel assays suggests that migrations over long distance are largely wind-assisted [56,57]. This “opportunistic” mode of dispersal, combined with the restricted distributions of most species of eucalypt, accords with short-range endemism previously described in Australian psyllids species [39,58]. In addition, the capacity of adults to acquire moisture (and possibly nutrition) from non-hosts may indicate that dispersing individuals can undertake multiple landings before reaching a suitable plant [10,46]. Their high reproductive rate may compensate for high mortality among dispersing individuals. Altogether, limited selective pressure exerted on the psyllid olfactory system for host location could be the main reason for the absence of chemotactic responses to host volatiles. In addition, psyllids associated with evergreen hosts as opposed to their host alternating counterparts may need to undertake greater microhabitat searching and selection at the between-host module level rather than finding of individual hosts.

To address this, we compared the resolution of host plant volatiles emitted by adult and juvenile leaves of *E. globulus* on the basis of existing physiological data for *C. eucalypti*. Specialization of the psyllid olfactory system has been proposed to have evolved via the dedication of their limited number of olfactory sensory neurons towards the detection of a few host-specific volatiles (to the detriment of more generic cues) as a way to overcome the absence of macro-structure in their antennal lobe [26] and decreased detection thresholds. The apparent lack of sensitivity of *C. eucalypti* antennae to predominant headspace compounds found in most eucalypts (e.g., 1,8-cineole, limonene, *α-*pinene, *γ-*terpinene and terpinyl acetate) appears to, at least partly, concur with such a hypothesis. Based on our results, preference for juvenile leaves would be governed solely by greater emissions of *β*-myrcene and avoidance of *β*-caryophyllene and *β*-ocimene. Unless *β*-caryophyllene and/or *β*-ocimene exert a repellent effect or act as dispersal cues to *C. eucalypti* in a similar way to *trans*-*β*-farnesene in aphids [59,60], the lack of sensitivity for specific compounds, other than *β*-myrcene, more abundantly emitted by juvenile leaves such as terpinyl acetate or limonene provide limited support for olfactory tuning in this species. 

In earlier studies, morphological differences between juvenile and adult leaves of *E. globulus* together with the presence of abundant non-structural epicuticular waxes were shown to greatly influence psyllid adhesion to leaf surfaces [61], probing [62,63], orientation behavior [64] as well as oviposition preferences [65]. Hence, it is possible that sensory modalities other than olfaction may play a more important role in the location of juvenile leaves by *C. eucalypti*. In addition, *Eucalyptus*-feeding psyllids, including the four species used in this study, have been shown to exploit visual cues such as leaf color and size to locate host leaves of the preferred age [15,16,17]. As a result, compelling evidence suggests that vision plays a predominant role in host location by these psyllids.

Olfactory cues may intervene after landing and mediate host selection and/or host module recognition. Psyllid olfactory sensilla have limited exposure to the air since they arise from the bottoms of cavities and do not protrude above the surface of the antennae. Their structure and location have been taken as evidence to suggest that they can only perceive static headspace compounds emanating from leaf surfaces of oleaginous plants [24]. It has also been suggested that the antennal olfactory apparatus of many Sternorrhyncha may have evolved to compensate for the absence of labial chemosensory sensilla [66]. Perhaps dynamic odors (e.g., those in olfactometer experiments) could be difficult for psyllids to orientate to as opposed to the static odors characteristic of the undisturbed boundary layer just above leaf surfaces. Such boundary layer odors could be more suited to perception by psyllid olfactory sensilla during antennation and may provide reliable post-landing cues [67]. For instance, *trans*-*β*-farnesene, present in the headspace of the wild potato (*Solanum berthaultii*), acts as a repellent to aphids (*Myrzus persicae*) after landing when insects make contact with the glandular trichomes from where it is exuded abundantly along with other sesquiterpenes [68]. In addition, ontogenic changes to epicuticular waxes and oil gland contents may also serve as post-alighting olfactory cues involved in host recognition [69,70,71]. Alternatively, olfactory cues may act as phagostimulants. Formic and acetic acids, derived from the degradation of terpenoids [72], have recently been shown to elicit strong electroantennographic responses and increased probing activities in psyllids [73,74]. Interestingly, sticky traps baited with acetic acid, also identified as a degradation product of the putative pheromone lignoceryl acetate, were found to significantly increase catches of *D. citri* under field conditions [75]. These findings highlight the importance of not only considering plant volatiles but also their degradation products when investigating plant–herbivore interactions. Nevertheless, such compounds, though intricately linked to the composition of HPVs, are very common in nature and are unlikely to be sufficiently specific to serve in isolation as olfactory cues for host finding or selection.

## 5. Conclusions

Our findings suggest that HPVs do not play a significant role in host finding in *Eucalyptus*-feeding psyllids but are more likely to intervene post-landing. While host finding may be partly—if not entirely—opportunistic, we suggest that active foraging is primarily driven by visual cues at the within-host canopy level. Olfactory cues probably act in conjunction with gustatory cues as phagostimulants and play a more important role in host recognition and host selection as appears to be the case with aphids [10,14,76] and Hemiptera more generally [77].

## Figures and Tables

**Table 1:** Headspace composition and volatile emission rates from leaves of *Eucalyptus* hosts of psyllids.

RT (min)	Tentative ID	*E. globulus* (Juvenile) n = 6		*E. globulus* (Adult) n = 8		*E. camaldulensis* n = 6		*E. kitsoniana* n = 7	
		Mean (ng/h) ^☨^	SE	Mean (ng/h) ^☨^	SE	Mean (ng/h) ^☨^	SE	Mean (ng/h) ^☨^	SE
6.63	𝛼-pinene	494	74	243	35	281	83	39	9
7.43	sabinene	11	5	6	2	3	1	3	1
7.53	*β*-pinene	39	5	13	2	13	5	4	1
7.79	*β*-myrcene *	372	137	84	26	14	6	16	6
8.03	*cis*-3-hexenyl acetate *	85	32	11	2	5	2	5	2
8.09	𝛼-phellandrene	95	70	11	3	1	1	0	0
8.45	p-cymene	92	24	25	2	11	9	34	23
8.56	limonene	1530	371	366	90	71	28	71	29
8.64	1,8-cineole	4269	1151	933	147	193	58	154	61
8.88	*trans*-*β*-ocimene *	328	166	1247	383	13	4	7	2
9.13	*𝛾*-terpinene	228	87	51	10	31	30	92	67
9.95	Linalool *	25	15	15	4	1	0	5	1
10.20	*trans-*4,8-dimethyl-1,3,7-nonatriene	410	191	221	64	1	0	0	0
11.39	𝛿-terpineol	15	9	1	1	0	0	0	0
11.55	terpinene-4-ol	39	21	4	1	1	0	3	2
11.81	𝛼-terpineol	260	152	10	3	2	1	3	1
12.65	a monoterpene acetate	20	6	0	0	0	0	1	1
12.80	nerol	15	12	0	0	0	0	0	0
13.13	geranial	8	6	0	0	0	0	0	0
14.03	methyl geranate	13	13	0	0	0	0	0	0
14.48	terpinyl acetate	760	453	13	6	1	0	18	8
14.90	𝛼-cubebene	31	9	5	2	0	0	0	0
14.98	𝛼-copaene	10	3	87	34	0	0	0	0
15.51	𝛼-gurjunene	139	55	29	9	0	0	2	1
15.73	*β*-caryophyllene *	334	112	664	141	7	3	2	1
16.03	aromadendrene	295	94	70	21	3	1	7	3
16.38	alloaromadene	148	76	37	9	1	0	2	1

RT: retention time; * indicates compounds that elicit the strongest SSR responses in *C. eucalypti*; ^☨^ Quantitation unit in ng n-tridecane equivalent/h.
